# Theory and application of TTFields in newly diagnosed glioblastoma

**DOI:** 10.1111/cns.14563

**Published:** 2024-03-13

**Authors:** Ao Yu, Juan Zeng, Jinhui Yu, Shuo Cao, Ailin Li

**Affiliations:** ^1^ Department of Radiotherapy, Liaoning Cancer Hospital & Institute, Cancer Hospital of China Medical University Cancer Hospital of Dalian University of Technology Shenyang China; ^2^ School of Graduate China Medical University Shenyang China; ^3^ Department of Oncology Shengjing Hospital of China Medical University Shenyang China

**Keywords:** alternating electric field, cancer therapy, glioblastoma, tumor treating fields

## Abstract

**Background:**

Glioblastoma is the most common primary malignant brain tumor in adults. TTFields is a therapy that use intermediate‐frequency and low‐intensity alternating electric fields to treat tumors. For patients with ndGBM, the addition of TTFields after the concurrent chemoradiotherapy phase of the Stupp regimen can improve prognosis. However, TTFields still has the potential to further prolong the survival of ndGBM patients.

**Aim:**

By summarizing the mechanism and application status of TTFields in the treatment of ndGBM, the application prospect of TTFields in ndbm treatment is prospected.

**Methods:**

We review the recent literature and included 76 articles to summarize the mechanism of TTfields in the treatment of ndGBM. The current clinical application status and potential health benefits of TTFields in the treatment of ndGBM are also discussed.

**Results:**

TTFields can interfere with tumor cell mitosis, lead to tumor cell apoptosis and increased autophagy, hinder DNA damage repair, induce ICD, activate tumor immune microenvironment, reduce cancer cell metastasis and invasion, and increase BBB permeability. TTFields combines with chemoradiotherapy has made progress, its optimal application time is being explored and the problems that need to be considered when retaining the electrode patches for radiotherapy are further discussed. TTFields shows potential in combination with immunotherapy, antimitotic agents, and PARP inhibitors, as well as in patients with subtentorial gliomas.

**Conclusion:**

This review summarizes mechanisms of TTFields in the treatment of ndGBM, and describes the current clinical application of TTFields in ndGBM. Through the understanding of its principle and application status, we believe that TTFields still has the potential to further prolong the survival of ndGBM patients. Thus,research is still needed to explore new ways to combine TTFields with other therapies and optimize the use of TTFields to realize its full potential in ndGBM patients.

## Graphical abstract

To explore the development direction of TTFields in the treatment of ndGBM, this paper summarizes the antitumor mechanisms of TTFields, including cell mitotic cycle interference, autophagy and apoptosis inducing, DNA damage repair inhibition, immune microenvironment activation, metastasis and migration reduction and blood–brain barrier permeability enhancement. In clinical applications, whether it is safe and feasible to advance the time of application of TTFields to a time concurrent with chemoradiotherapy has been preliminarily explored. After proper adjustment of the PTV and scalp protection, wearing electrode patches during radiotherapy is safe and feasible. In addition, TTField therapy shows potential in combination with immune checkpoint inhibitors, tumor vaccines, antimitotic drugs, and PARP inhibitors as well as the potential for application in patients with subtentorial glioma.

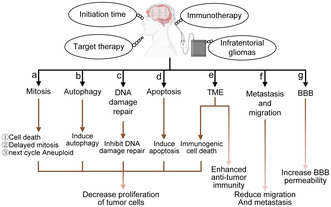

## 1 INTRODUCTION

Glioblastoma (GBM) is the most common primary brain malignancy. Tumor‐treating fields (TTFields) therapy is the first treatment since 2005 to significantly improve median progression‐free survival (mPFS) and overall survival (mOS) in patients with GBM. TTFields constitutes a noninvasive antitumor approach involving insulated transducer arrays placed directly on the skin in the tumor region that produce low‐intensity (1–3 V/cm), medium‐frequency (100–300 kHz) alternating electric fields for antitumor purposes in local areas. TTFields was approved by the FDA for use in patients with newly diagnosed GBM (ndGBM) in 2015 and has been included in treatment guidelines. Herein, the mechanism of action of TTFields is revealed: mitosis interference, autophagy induction, DNA damage repair inhibition, apoptosis induction, blood–brain barrier (BBB) permeability elevation, cancer cell migration and metastasis suppression, and immunosuppressive microenvironment alteration. Finally, the currently used TTFields application for ndGBM patients is described, and future directions for development are proposed based on the mechanism of action of TTFields, including directions regarding the timing, indications, and details of TTField applications alone and in combination with immunotherapy and targeted therapy.

## 2 MECHANISM OF ACTION OF TTFIELDS

### 2.1 TTFields interfere with mitosis

TTFields affect mitosis only in actively proliferating tumor cells; normal nerve cells are considered unaffected because they divide slowly.^1^ The antimitogenic effect of TTFields is accomplished by electric field force‐mediated dipole rearrangement and dielectrophoretic effects. Cells contain many charged particles and polar molecules, which can generate their own electric fields and can also react to external electric fields. During tumor cell proliferation, α/β tubulin dimers are arranged by their own electric fields to form spindles, and the septin2‐6‐7 complex is positioned to form a cleavage furrow and contractile ring. TTFields act mainly on these two high‐dipole‐moment proteins in tumor cells.^2^

First, during mitotic metaphase, tubulin is disturbed by uniform alternating electric field forces generated by TTFields. Tubulin oscillates and spins, disrupting the stability of microtubule heterodimeric protein polymerization and leading to spindle assembly errors and abnormal geometric shapes. Eventually, these effects cause delayed mitosis, abnormal mitotic exit in tumor cells, decreased cell proliferation, and aneuploid cell formation.^3^, ^4^ Next, during mitotic anaphase, electric field forces interfere with the movement and binding of the septin protein, inhibiting its midline localization and function. The contractile elements of the cell membrane spread in a disordered manner throughout the cell, which eventually undergoes violent ectopic contraction, causing cell membrane blebbing.^5^ Finally, during mitotic telophase, the cell acquires an hourglass shape, and the electric field lines are highly clustered at the cleavage furrow, generating an uneven alternating electric field that exerts a dielectrophoretic effect on the cytoplasm; in this process, charged macromolecules and organelles are propelled toward the neck of the daughter cell that will soon separate. The cell membrane pressure increases, and the cell ruptures and dies.^6^

In addition, through transcriptomic and proteomic analysis, Xu et al. showed that TTFields can reduce CDK2‐AS1 expression, thereby reducing mRNA stability and CDK2 expression and ultimately resulting in G1 phase cell cycle arrest and interfering with tumor cell proliferation.^7^

### 2.2 TTFields induce autophagy in tumor cells

Few cells treated with TTFields stop dividing and die during mitosis; instead, most cells die during interphase in the next division cycle.^8^ TTField‐induced autophagy results from abnormal mitosis rather than M phase arrest. TTFields can lead to abnormal chromosome separation, which can result in the formation of aneuploid cells. Cellular aneuploidy is associated with the activation of genes related to autophagy regulatory factors.^9^ A low rate of chromosome segregation errors (resulting in the formation of fewer aneuploid cells) promotes tumorigenesis, while a high rate (resulting in the formation of many aneuploid cells) leads to cell death and tumor suppression.^10^

Silginer used TTFields to treat glioma cells in vitro and found that TTFields killed tumor cells through autophagy.^11^ When TTFields was used to treat glioma cell lines, the treated cells exhibited autophagy‐related changes, such as increased lysosomal volume and cell granularity, as well as increased conversion of light chain 3 (LC3) to LC3‐ii, a marker of activated autophagy. Subsequently, autophagosomes bind to lysosomes to complete autophagy. TTField‐induced autophagy was also associated with the activation of AMPK phosphorylation and blockade of the miR‐29b‐Akt2 pathway.^12^, ^13^ After TTField treatment, the expression of autophagy‐related genes in glioma cells was upregulated by approximately 2‐fold.^13^

### 2.3 TTFields inhibit DNA damage repair

TTFields can inhibit DNA damage repair. The interference of TTFields with DNA damage repair was confirmed by alkaline comet assay and γH2AX analysis, a common method for detecting double‐stranded DNA damage.^14^ This mechanism is a key reason why TTFields can be used in combination with antitumor therapies such as radiotherapy (RT) and chemotherapy. More significant and longer‐lasting DNA damage is observed in ionizing radiation (IR) + TTField‐treated GBM cell lines than in those treated with IR alone, and this damage is accompanied by an increase in γH2AX foci.^15^ Whether TTFields is applied before or after RT, cancer cells are more sensitive to radiation.^16^ Similarly, TTFields increases the cytotoxicity of bleomycin, a DNA‐break inducer, toward glioma cells.^14^ In mice with pleural mesothelioma, TTFields combined with cisplatin and pemetrexed significantly reduced tumor volume and increased the number of γH2ax foci.^17^, ^18^

What is the molecular mechanism underlying this effect? TTFields induce DNA replication pressure, which slows replication, reduces replication accuracy, and increases R‐loop formation. R‐loops are markers of replication stress, and their accumulation at DNA damage sites can hinder homologous recombination repair.^19^ Mumblat observed that after treating pleural mesothelioma cells with TTFields, BRCA1 gene expression was significantly downregulated.^17^ Similarly, after glioma cell lines were treated with TTFields, downregulation of BRCA2 gene expression was observed with increased numbers of γH2AX foci. BRCA1 and BRCA2 play an important role in maintaining the fidelity of DNA replication by mediating homologous recombination repair, and downregulation of BRCA gene expression can inhibit DNA double‐strand repair.^20^

PARP is a protein involved in DNA repair. PARP inhibitor treatment induces cancer cell death in patients with BRCA mutations or defects.^21^ The downregulation of BRCA gene expression induced by TTFields provides the theoretical basis for the combination of TTFields and PARP inhibitors. Kim et al. found that caspase‐3 is a protease that specifically cleaves PARP1 and that TTFields therapy leads to caspase‐3 activation and PARP‐1 cleavage in glioma cell lines.^15^ On the basis of TTField‐induced BRCA1 downregulation combined with increased PARP cleavage, this phenomenon results in effects similar to those of PARP inhibitors.

### 2.4 TTFields induce apoptosis of cancer cells

TTFields inhibits the proliferation and induces the apoptosis of GBM cells.^7^ Caspase‐3 is a key protease in apoptosis, and TTFields can mediate tumor cell apoptosis by activating caspase‐3.^22^ TTFields can also induce apoptosis in a caspase‐independent manner. High circMMD expression in GBM leads to poor prognosis, and TTFields intervention can reduce circMMD synthesis. The reduction in circMMD promotes the FUBP1–FIR interaction, thereby reducing DVL1 transcription; it also promotes miR‐15b‐5p‐mediated FZD6 degradation. Decreased DVL1 and FZD6 expression inhibits Wnt/β‐catenin pathway activation. Finally, TTF‐mediated apoptosis is increased, and GBM proliferation is inhibited.^23^

The combination of TTFields with sorafenib, TMZ + lomustine, RT, or hyperthermia enhances the apoptosis induced by TTFields.^15^, ^20^, ^24^, ^25^

### 2.5 TTFields alter the tumor immune microenvironment

GBM is defined as a “cold” tumor. The immune cell components in the GBM tumor microenvironment (TME) are complex and highly heterozygous, and the TME contains many infiltrating microglia,^26^ which can lead to an immunosuppressive microenvironment.^27^ TTFields can convert an immune “cold” tumor into a “hot” tumor.

TTField‐mediated activation of glioma immunity has been gradually revealed. Tumor cells undergo immunogenic cell death (ICD) under exposure to external stimuli, during which damage‐associated molecular patterns (DAMPs), including calreticulin (CRT), HSP 70/90, HMGB1, ATP, TNF‐α, ROS, and IFNs α and β, activate the immune system.^28^, ^29^, ^30^ TTFields can cause ICD through various mechanisms. In an animal experiment, TTField‐treated lung metastases were found to have greater CD45+ T‐cell infiltration than lesions in the sham controls. CD45+ T cells can induce TNF‐α production, which can lead to ICD.^30^ After TTField‐induced cell death, HMGB1 and ATP are released, and cell membrane surface exposure to CRT and CD45+ lymphocyte recruitment are increased; these molecules are markers of ICD.^31^ Additionally, after TTField treatment, the cGAS/STING and AIM2/caspase‐1 pathways were activated in GBM cell lines. T‐cell activation and clonal expansion as well as increased secretion of proinflammatory factors such as IL‐6 and INF‐1 are observed. These effects in turn enhance antitumor immunity and lead to ICD.^32^ TTFields can also upregulate reactive oxygen species (ROS) production and induce ICD.^15^

TTFields promote dendritic cell (DC) maturation Microtubule stability disruption results in increased release and activation of guanine nucleotide exchange factor‐H1 (GEF‐H1),^33^ which accelerates DC maturation and promotes antigen presentation. Moreover, increased cell membrane expression of MHCII, CD40, and CD80 molecules has been found on DCs, promoting DC maturation.^34^ Regarding macrophages, TTFields activates the macrophage‐specific immune response by modulating the p38 MAPK and NF‐kB pathways.^35^ Treating macrophages induces the polarization of M2‐type macrophages to the M1‐type.^36^

RT and TMZ can cause immunosuppression. After TTFields is added to the standard chemoradiotherapy regimen, the number of infiltrating lymphocytes in the TME of ndGBM patients significantly increases, and this increase is accompanied by signs of activation.^37^ Immunotherapy combined with TTFields may better enhance immunity, as the numbers of classical and plasmacytoid DCs increase by 2.26‐fold and 5.2‐fold, respectively, when TTFields are combined with pembrolizumab and TMZ for the treatment of ndGBM patients, suggesting that anti‐PD‐1 therapy may enhance the immune effect induced by TTFields.^38^

### 2.6 TTFields reduces glioma cell metastasis and migration rates

One reason for the short patient survival time and high recurrence rate of GBM is the highly metastatic and aggressive nature of GBM, and TTFields can prevent the metastasis and spread of primary tumors.^30^

Cell migration and wound healing assays revealed significant decreases in the migration and invasion of U87, U373, A172, LN18, and LN229 glioma cells treated with TTFields as well as a significant reduction in the migration of T325 and ZH161 glioma stem cells.^11^, ^39^, ^40^ Epithelial–mesenchymal transition (EMT) enhances cell motility and migration ability. Epithelial markers are upregulated, mesenchymal markers are downregulated, and EMT‐related genes are dysregulated in TTField‐treated cells. SHH/GLI1 signaling pathway activation is involved in EMT and is closely related to cancer cell invasion and metastasis.^41^ The SHH pathway is activated in GBM.^42^ Primary cilia are abundant on the surface of the glioma cell membrane, forming the structural basis of SHH/GLI1 signal transduction and pathway activation.^43^ TTFields can destroy primary cilia, thus affecting SHH pathway activity and interfering with glioma cell invasion and metastasis.^44^ Angiogenesis is also closely related to tumor metastasis. TTFields inhibits vascular endothelial cell growth; downregulates HIF1α, VEGF, and MMP2/9 expression; and inhibits neovascularization. TTField‐mediated inhibition of EMT and neovascularization is associated with PI3K/AKT/NF‐κB signaling pathway downregulation.^40^, ^45^

Focal adhesions and the actin network surrounding cells prevent cell invasion and metastasis. A172 and LN229 glioma cells treated with TTFields exhibited an increased area and number of focal adhesions and a dense surrounding actin network. The reason for these effects is related to increased activation of GEF‐H1 caused by the effect of TTFields on microtubules, which results in activation of the RhoA/ROCK pathway.^33^

### 2.7 TTFields disrupts the BBB

What is the status of the BBB in GBM patients? Based on imaging and surgical experience, Sarkaria et al. proposed that part of the tumor region in GBM is protected by an intact BBB, which can lead to uneven drug distribution.^46^ Yuan Xie et al. found a heterogeneous status of BBB endothelial cells, which resulted in damage to parts of the BBB while other parts remained intact.^47^ Substances cross the BBB via two main pathways: the paracellular pathway, mediated by the tight junction (TJ) protein claudin‐5, and the transcellular pathway, mediated by endocytosis. Although TMZ has been suggested to be an effective anti‐GBM drug, other drugs with potential potency against GBM are ineffective due to the BBB. Immunoreactive substances and immune cells cannot easily cross the BBB, resulting in unsatisfactory efficacy of glioma immunotherapy.^48^ Strategies to disrupt the BBB should be considered in the design of clinical trials for GBM patients.

Sharabi et al. performed experiments applying low‐voltage pulses (5–100 V) to an external human BBB model. While the mechanism was unclear, low‐voltage pulsed electric fields (PEFs) briefly breached the BBB by affecting the paracellular pathway.^49^ Salvador et al. explored the effects of TTFields on the BBB and found that TTFields resulted in reversible BBB opening.^50^ In in vitro experiments, they used TTFields to treat mouse cerebellar microvascular endothelial cells. Immunofluorescence analysis showed that the TJ protein claudin‐5 in endothelial cell connections relocalized from the cell membrane to the cytoplasm. In cancer cells, TTFields can activate the Rho/Rho kinase pathway, through which a threonine of claudin‐5 is phosphorylated, thereby interfering with claudin‐5 binding to other TJ‐anchored proteins and leading to increased BBB permeability.^51^ Neither gadolinium contrast nor paclitaxel can cross the BBB. In vivo experiments have shown that TTFields increases gadolinium accumulation in the rat brain and, in combination with paclitaxel, permits the effects of paclitaxel and reduces both the tumor volume and tumor cell proliferation in rats. An in vitro 3D coculture model of the human BBB has been constructed using human brain microvascular endothelial cells (HBMVECs) and immortalized human pericytes.^52^ TTFields increased BBB permeability in this model by altering the location of intracellular TJ proteins in HBMVECs.

**FIGURE 1:**
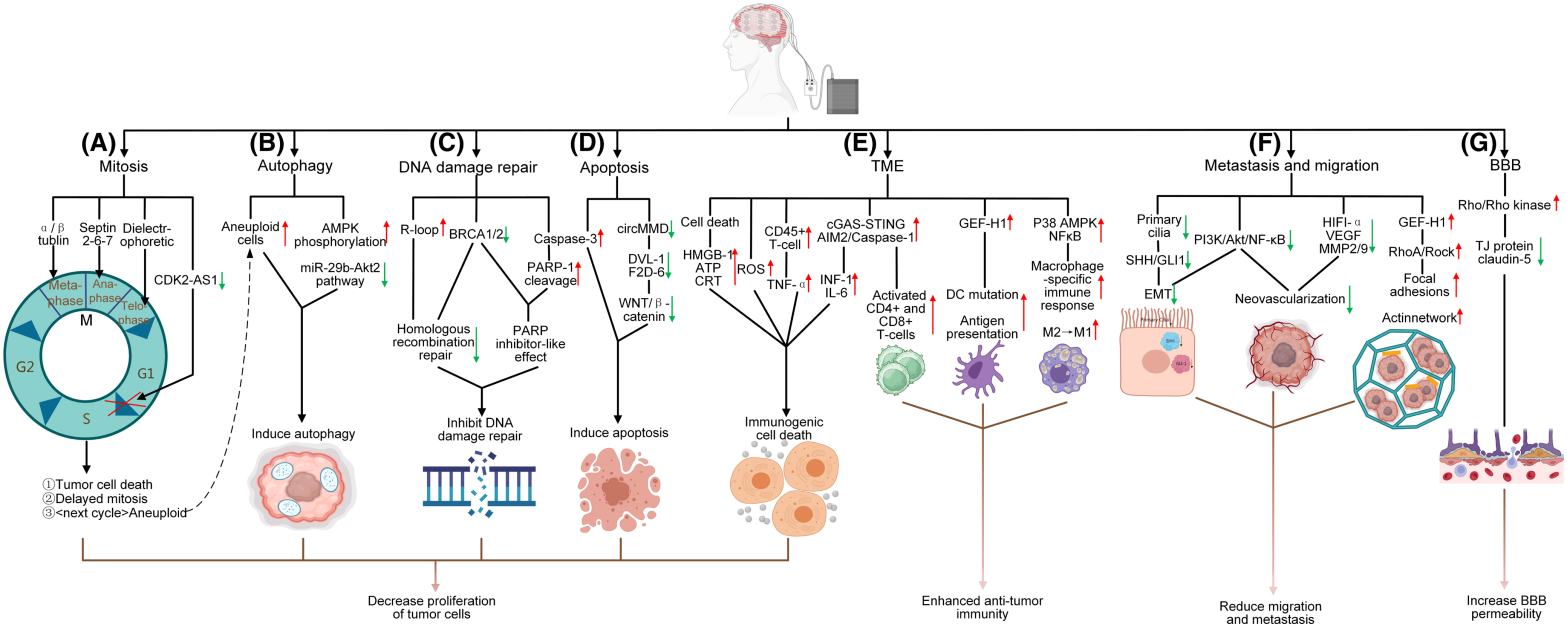
Mechanism of action of TTFields against glioblastoma. ↑ upregulated, ↓ downregulated. (A). TTFields perturbs mitosis at metaphase, anaphase, and telophase by interfering with α/β tubulin dimers, the septin2‐6‐7 complex, and cytosolic electrophoresis, and they also block the process of mitosis by downregulating CDK2‐AS1. Eventually, these changes result in tumor cell death, delayed mitosis, and increased aneuploidy. (B). TTField‐induced autophagy is associated with increases in the number of aneuploid cells, activation of AMPK phosphorylation, and inhibition of the miR‐29b‐Akt2 pathway. (C). TTFields can increase R‐loop formation and downregulate the BRCA pathway, thereby hindering homologous recombination repair. In addition, they exert a PARP inhibitor‐like effect by downregulating the BRCA pathway and increasing PARP cleavage. (D). TTFields induces tumor cell apoptosis by downregulating DVL‐1 and F2D‐6 or upregulating caspase‐3. (E). TTFields caused cell death, resulting in HMGB‐1 and ATP release and CRT exposure. In response to TTField stimulation, ROS levels increase, and upregulation of cGAS‐STING and the AIM2/caspase‐1 pathway leads to upregulation of IL‐6 and INF‐1. Eventually, these factors lead to immunogenic cell death. TTFields induce T‐cell activation through upregulation of cGAS‐STING and AIM2/caspase‐1. By upregulating the GEF‐H1, P38 AMPK, and NFkB pathways, TTFields promotes the antitumor function of DCs and macrophages. (F). TTField‐mediated inhibition of EMT and neovascularization, expansion of the area of focal adhesions, and creation of a dense surrounding actin network together interfere with tumor cell invasion and metastasis. (G). TTFields activate the Rho/Rho kinase pathway, thereby interfering with claudin‐5 binding to other TJ‐anchored proteins and leading to increased BBB permeability.

Figure 1 summarizes the mechanism of action of TTFields against glioblastoma. Table 1 lists the pivotal preclinical studies of TTFields in GBM.

**TABLE 1:** Pivotal preclinical studies of TTFields in GBM.

Study	Design	Outcomes	Ref
Giladi *et al*. 2015	In vitro	TTFields interfere with tubulin polymerization and prevent normal spindle assembly	[8]
Gera *et al*. 2015	In vitro	TTFields cause abnormal mitosis by interfering with the Septin complex	[5]
Kim *et al*. 2016	In vitro	TTFields interfere with EMT, downregulate VEGF, HIF1‐α and MMP2/9, and inhibit the transcriptional activity of NF‐kB, thereby interfering with the invasion and metastasis of GBM cells	[40]
Silginer *et al*. 2017	In vitro	TTFields induce autophagy in glioma cells and hindered their migration	[11]
Shteingauz *et al*. 2018	In vitro	TTFields induced AMPK‐related autophagy in glioma cells	[12]
Kim *et al*. 2019	In vitro and in vivo	Autophagy induced by TTFields is closely related to GBM cell death	[13]
Karanam *et al*. 2019	In vitro	TTFields increase the stress of DNA replication and interfere with DNA damage repair	[19]
Park *et al*. 2019	In vitro	TTFields activate macrophage‐specific antitumor immunity via the NK‐κB/MAPK pathway	[35]
Voloshin *et al*. 2019	In vitro and in vivo	TTFields promote DC recruitment and maturation and induce immunogenic cell death	[31]
Voloshin *et al*. 2020	In vitro and in vivo	TTFields interfere with tumor cell motility by regulating microtubule and actin dynamics	[33]
Oh *et al*. 2020	In vitro	TTFields inhibit EMT and downregulate the expression of MMP2 and VEGF, thus inhibiting tumor cell migration and invasion	[39]
Wu *et al*. 2020	In vivo	The expression of caspase‐3 increased and the tumor volume decreased after TTFields treatment of glioma	[22]
Salvador *et al*. 2022	In vitro and in vivo	TTFields interfere with the binding of claudin‐5 to other tight junction proteins by activating the Rho/Rho kinase pathway, resulting in increased BBB permeability	[50]
Xu *et al*. 2022	In vitro	Decreased CDK2‐AS1 expression resulted in cell cycle arrest in G1 phase	[7]
Chen *et al*. 2022	In vitro and in vivo	TTFields activate antitumor immunity by activating GAS/STING and AIM2/caspase‐1 pathways	[32]
Fishman *et al*. 2023	In vitro	TTFields downregulate the FA‐BRCA pathway and increase chemotherapy‐induced DNA damage in GBM cells	[20]
Xu *et al*. 2023	In vitro	TTFields can reduce circMMD synthesis and increase GBM cell apoptosis.	[21]
Salvador *et al*. 2023	In vitro	TTFields alter the location of endothelial tight junction proteins to increase BBB permeability	[52]

Abbreviations: BBB, Blood–Brain‐Barrier; DC, dendritic cell; EMT, Epithelial‐Mesenchymal Transition; GBM, glioblastoma; HIF1‐α, Hypoxia‐inducible Factor‐1α; MMP2/9, matrix metallopeptidase 2/9; RT, radiotherapy; TMZ, temozolomide; TTFields, treatment method called tumor treating fields; VEGF, vascular endothelial growth factor.

## 3 USE OF TTFIELDS TO TREAT ndGBM PATIENTS: PRESENT AND FUTURE

GBM is highly malignant, and the recurrence rate is close to 100%. It is well known that tumor recurrence is associated with a poorer prognosis and shorter survival time. Therefore, improving the effect of initial treatment and delaying tumor recurrence are crucial to improve the survival of ndGBM patients. We list the pivotal clinical studies on TTFields for ndGBM (Table 2).

**TABLE 2:** Pivotal clinical studies of TTFields in ndGBM.

Study	Region	Phase	Treatments and Sample size	Outcomes	Ref
EF‐07	Czech Republic	Pilot clinical trial	TMZ/TTFields 10	PFS:155 weeks OS:more than 39 months	[53]
EF‐14	Global	Randomized phase III clinical trial	TMZ/TTFields arm:466 TMZ arm:229	mPFS:6.7 vs. 4.0 months mOS:20.9 vs. 16.0 months	[54]
Bokstein *et al*. 2020	Israel	Pilot study	Concurrent TTFields/RT/TMZ (patch removed) arm:10	mPFS:8.9 months	[55]
Miller *et al*. 2022	US	Pilot study	Concurrent TTFields/RT/TMZ (patch retained) arm:30	Safe and tolerable	[56]
Lazaridis *et al*. 2022	Germany	Retrospective study	TMZ/Lomustine/TTFields≥8 weeks arm:22 TMZ/Lomustine/TTFields<8 weeks arm:48	mPFS: 21.5 vs. 11.2 months	[57]
PriCoTTF	Germany	Phase I/II	Concurrent TTFields/RT/TMZ (patch retained) arm:33	Safe and tolerable	[58]
2‐THE‐TOP	US	Phase II	TMZ/Pembrolizumab/TTFields arm:26 TMZ/TTFields arm:26	mPFS:12.0 vs. 5.8 months mOS:24.8 vs. 14.7 months	[59]
Vymazal *et al*. 2023	Czech Republic	Retrospective study	TMZ/TTFields arm:55 TMZ arm:54	mPFS:19.75 vs. 12.45 months mOS:31.67 vs. 24.80 months	[60]

Abbreviations: mOS, median overall survival; mPFS, median progression‐free survival; ndGBM, newly diagnosed glioblastoma; RT, radiotherapy; TMZ, temozolomide; TTFields, treatment method called tumor treating fields.

### 3.1 The new Stupp regimen

Stupp published the standard of care for ndGBM patients: maximum safe resection followed by RT + TMZ. This was a milestone in ndGBM treatment. Patients treated with the Stupp regimen have an mOS of 14.6 months.^61^ In the face of an unsatisfactory prognosis, people continue to explore treatments for GBM, and TTFields is a promising therapy. In 2004, a pilot clinical trial using TTFields for GBM (EF‐07) was initiated and enrolled 10 recurrent GBM (rGBM) and 10 ndGBM patients. After surgery and RT + TMZ treatment, the 10 ndGBM patients were treated with TTFields+TMZ and achieved a PFS time of 155 weeks and an OS time of more than 39 months. The only adverse event was contact dermatitis at the electrode contact site. The EF‐07 trial initially confirmed the therapeutic advantages and safety of TTFields combined with the Stupp regimen in ndGBM.^53^

Ten years later, the results of EF‐14, a randomized, phase III clinical trial that randomly assigned patients after completion of chemoradiotherapy to receive TMZ + TTFields or TMZ monotherapy, were encouraging. After the return of the interim results, the FDA approved the use of TTFields as a treatment for ndGBM. In 2017, the final EF‐14 results were published. In patients treated with the Stupp regimen+TTFields vs. those treated with the Stupp regimen, the mPFS time was 6.7 vs. 4.0 months, and the mOS time was 20.9 vs. 16.0 months.^54^ Due to its excellent efficacy, the Stupp regimen+TTFields became the new standard treatment regimen, known as the new Stupp regimen. The new Stupp regimen showed a more pronounced benefit in the Asian subgroup than the Stupp regimen, with increased PFS (6.2 vs. 4.2 months) and OS (27.2 vs. 15.2 months) times.^62^ Elderly ndGBM patients are more difficult to treat and have a poorer prognosis than younger patients. The new Stupp regimen was well tolerated in elderly patients and, compared with the Stupp regimen, increased PFS (6.5 vs. 3.9 months) and OS (17.4 vs. 13.7 months) times.^63^ Patients with high TTField wear compliance (daily treatment >22 h) showed a greater survival benefit than those with low compliance, with a 5 years OS of 29.3%.^64^

### 3.2 Potential applications of TTFields

Currently, TTFields for ndGBM patients is administered after chemoradiation. As a therapy with great potential, more aspects of TTField application are being explored, such as the treatment time, combination with targeted therapy or immunotherapy, and the possibility of treating subtentorial glioma.

**FIGURE 2:**
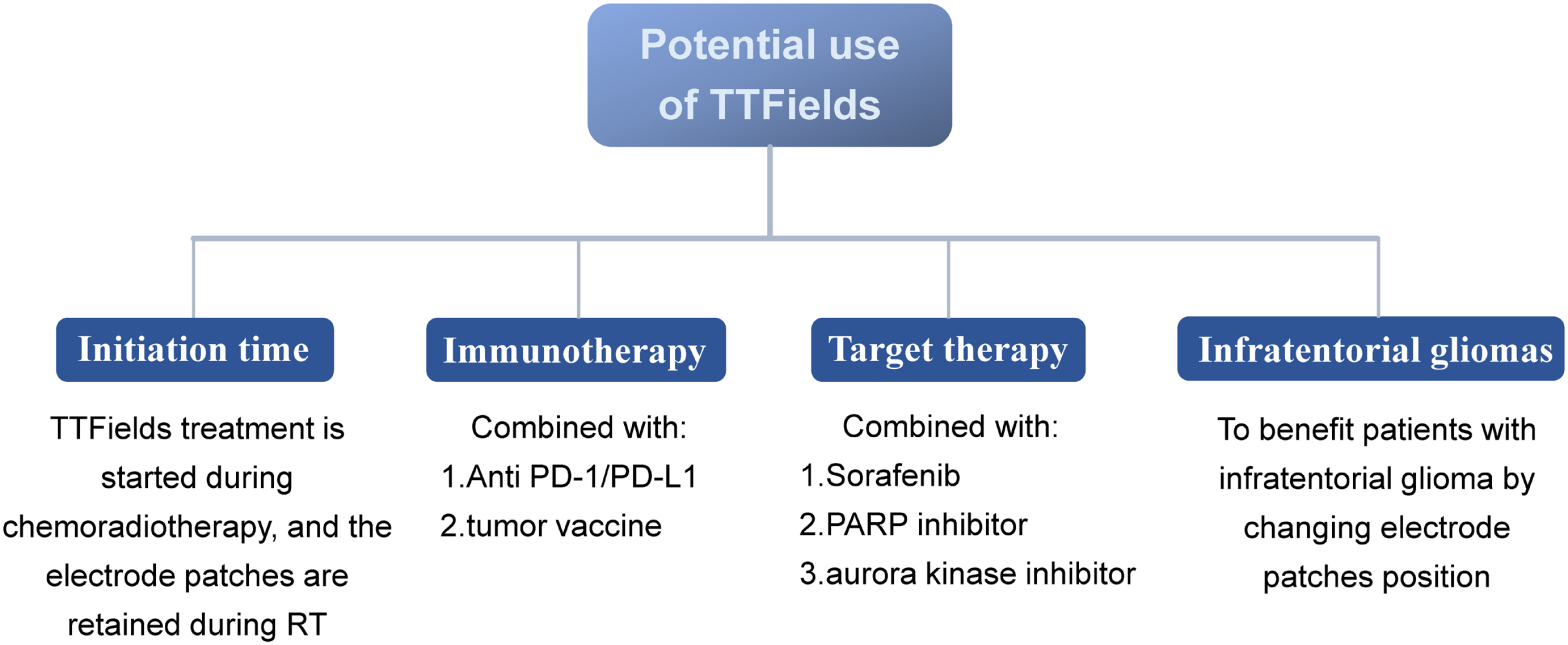
Potential applications of TTFields.

The potential applications of TTFields are summarized in Figure 2.

#### 3.2.1 Timing of TTField treatment initiation

Bokstein treated ndGBM patients with TTFields/RT/TMZ followed by adjuvant TMZ/TTFields, and the patients had an mPFS time of 8.9 months. TTFields did not increase RT‐ or TMZ‐related toxicity, and no TTField‐related adverse events except for grade 1–2 skin toxicity were observed, indicating that adding TTFields in the chemoradiotherapy phase is safe and feasible, with good preliminary efficacy.^55^ In this study, the electrode patches were removed before each RT session. Electrode patches are expensive, and their replacement each time is tedious, requiring more than 1 h and extending the interval between radiation and TTFields therapy. According to clinical experience, shortening the interval between RT and TTFields therapy can improve efficacy. Therefore, the follow‐up PriCoTTF and EF‐32 trials preserved the electrode patch. The PriCoTTF trial was a phase I/II study in 33 ndGBM patients. TTFields therapy continues throughout RT + TMZ and adjuvant TMZ for a total of 9 months. The latest results reported in 2022 indicated that concurrent treatment with TTFields and RT was well tolerated by patients and that grade ≥3 adverse skin events occurred in only two cases.^58^ In 2020, Novocure started a randomized prospective open‐label phase 3 trial (EF‐32) in which patients with ndGBM were treated with TTFields in combination with RT and TMZ to evaluate whether concurrent initiation of TTFields and postoperative RT improves clinical outcomes over those who achieved the standard treatment. The study is scheduled to enroll 950 patients at 129 sites worldwide; the primary endpoint is patient OS, with an estimated primary completion time of August 2024.

Retention of electrode patches is often accompanied by concerns about RT target dose coverage and scalp damage. Indeed, researchers have observed dose increases of up to 8.5% within 2 mm of the scalp when RT is performed with electrode patch retention, which could lead to scalp rupture.^65^ Therefore, protecting the scalp as an organ at risk (OAR) seems to be a good option. Ryan Miller conducted a related study involving the application of RT/TMZ/TTFields with scalp preservation and electrode patch retention. Planning target volume (PTV) coverage took precedence over scalp dose limits when necessary. At the median follow‐up of 15.2 months, grade 1/2 adverse skin events occurred in 83.3% of patients. These results confirm that this treatment modality is safe and tolerable.^56^ Guberina found that the dose distribution within the clinical target volume (CTV) is not significantly clinically compromised by electrode patch retention, only resulting in less than a 2% decrease in D95 for the CTV, typically below 1%.^65^ However, patch retention can lead to changes in the patient's position, bringing uncertainty to the PTV. Therefore, image‐guided RT (IGRT) is recommended. For patients undergoing non‐IGRT, a PTV dilation boundary of 5 mm is recommended to improve the efficacy of RT because in the plans with 3 mm and 4 mm CTV expansion, the coverage of prescription dose in CTV decreased to 92%.^66^ These studies confirm that RT can be delivered with electrode patch preservation after PTV adjustment and scalp protection.

#### 3.2.2 TTFields in combination with immunotherapy

Notable progress has been made in the therapeutic use of PD‐1 and PD‐L1 checkpoint inhibitors in many tumors, and PD‐L1 expression has been detected in 88% of ndGBM samples.^67^ However, the use of immune checkpoint inhibitors in the treatment of ndGBM did not improve survival in two randomized multicenter phase III trials, Checkmate498 and Checkmate548.^68^, ^69^ Could the addition of TTFields improve the performance of ndGBM immunotherapy? 2‐THE‐TOP was a phase II prospective single‐arm open‐label clinical trial in which the enrolled ndGBM patients were treated with pembrolizumab, TTFields, and TMZ after completing concurrent chemoradiotherapy. Twenty‐six patients from the 2‐THE‐TOP study were paired with 26 patients from the historical EF‐14 study treated with TTFields + TMZ. After the final analysis, patients in the 2‐THE‐TOP study vs. patients in the historical EF‐14 cohort had mPFS times of 12.0 vs. 5.8 months and mOS times of 24.8 vs. 14.7 months and tolerated the triple therapy well.^59^

Cancer vaccines such as peptide vaccines and DC vaccines have shown great promise in glioma immunotherapy. In one ongoing single‐arm single‐center open‐label phase I clinical trial that included 13 ndGBM patients, a poly‐ICLC tumor vaccine was administered in the TMZ and TTField phases after chemoradiotherapy to explore the safety and potential clinical benefits of the tumor vaccine combined with TTFields. The primary outcome measure of this trial is dose‐limiting toxicity, and the secondary outcome measures are the toxicity grade, PFS, OS, and overall response rate (ORR). DC vaccine therapy starts with the isolation of DCs from the patient's blood followed by exposure of the DCs to a tumor sample from the patient and activation of the DCs. The activated DCs are then transfused back into the patient to activate the immune system and kill cancer cells. In a multicenter prospective phase III trial, the DCVax‐L vaccine was added to the chemoradiotherapy and adjuvant TMZ treatment phase of standard care (surgery/RT/TMZ). The results showed significantly better OS in ndGBM patients treated with DCVax‐L than in those treated with standard therapy. The risk of death at any time point was reduced by 20%, and this survival benefit increased over time. As mentioned above, TTFields can recruit and activate DCs; therefore, does combining DCVax‐L with TTFields produce a more powerful antitumor effect? In this trial, eight patients were treated with TTFields after tumor recurrence. Four patients (50.0%) were treated with DCVax‐L and survived for 22.6–72.7 months after randomization. As controls, four patients (50.0%) stopped DCVax‐L therapy and survived for between 8.9 and 29.2 months after randomization.^70^ These preliminary results suggest that TTFields combined with DCVax‐L is a beneficial treatment regimen, but larger trials are needed to verify its safety and efficacy.

#### 3.2.3 TTFields combined with targeted therapy

Sorafenib is a multitarget antitumor agent, and one study has demonstrated the potential of TTFields to increase the sorafenib sensitivity of liver cancer cells.^71^ As mentioned above, PARP inhibitors have potential for combined application with TTFields. A phase II trial (NCT04221503) evaluating niraparib in combination with TTFields in rGBM patients is ongoing. We also believe that combination treatment with PARP inhibitors and TTFields can benefit ndGBM patients and shows great potential for future applications. In a case report, a 57‐year‐old female patient with astrocytoma with a GBM molecular signature, low MGMT promoter methylation, and wild‐type IDH was treated with the Stupp regimen, antiangiogenic agents, and methotrexate. However, the tumor progressed rapidly during the treatment. After adjustment of the treatment plan to RT combined with TTFields, niraparib, and anlotinib, the tumor was effectively controlled, and the patient's condition became stable, which suggests that TTFields, PARP inhibitors, and anlotinib may have a synergistic effect on tumor control during RT for rapidly progressing GBM.^72^

Aurora kinases play important roles in mitosis and participate in events such as spindle assembly, chromosome separation, and cytokinesis. There are currently no commercial aurora kinase inhibitors, but numerous studies have been conducted to indicate that aurora kinases are promising targets. In an in vitro experiment, compared with either treatment alone, combination treatment with AZD1152 (an aurora B kinase inhibitor) and TTFields was found to significantly reduce the numbers of primary cultured ndGBM and rGBM cells. This suggests that the combination of TTFields and aurora kinase inhibitor drugs can further improve antitumor efficacy.^73^

#### 3.2.4 Evaluation of TTFields feasibility in infratentorial glioma

The prognosis of subtentorial glioma patients is often poor. Due to the placement of the electrode patch, a major limitation of TTFields is that it can only be used to treat supratentorial tumors. However, one study showed that it was possible to personalize treatment planning based on specific placement of the electrode patch.^74^ Therefore, researchers have tested other electrode patch locations to explore whether TTFields can be used to treat subtentorial tumors. Lok conducted a finite element model trial and found that compared with the coverage provided by current electrode patch placement sites for supratentorial tumors, the electric field coverage of cerebellar tumors was improved by placing electrode patches on the crown, bilateral posterolateral occipital bones, and posterior aspect of the neck.^75^ In one trial, TTFields was applied to a model of an adult male head; electrode patches were attached to the top of the head, the posterolateral occipital bone on both sides of the skull, and the posterior aspect of the neck. In the infratentorial region, the average electric field intensity in the vertical and horizontal directions was 1.7 V/cm and 2 V/cm, respectively, indicating that the electric field intensity requirement (1–3 V/cm) for TTField treatment of infratentorial glioma can be met by changing the position of the electrode patch.^76^ An ongoing trial (NCT05310448) is being performed to evaluate TTFields in brainstem GBM patients.

While the inherent properties of TTField therapy make it clinically versatile, there are many unresolved clinical issues regarding TTFields. We list the ongoing clinical studies on TTFields for ndGBM (Table 3).

**TABLE 3:** Ongoing trials of TTFields in glioblastoma as of 24 September 2023.

NCT Number	Characteristic	Arms	Treatment	Sample Size	Primary outcome measures	Duration
NCT04397679	Phase I	1	RT, TTFields TMZ, Chloroquine	10	Proportion of patients develop dermatitis	3 years
NCT04218019	Phase I	2	TTFields	68	AE, SCTR	2 years
NCT03705351	Phase I	1	TTFields, TMZ, RT	7	AE	6 years
NCT03477110	Phase I	1	TTFields, TMZ, RT	35	Discontinuation rate due to skin toxicity	3 years
NCT03194971	Phase II	2	TTFields	20	States of mitotically cells	7 years
NCT04757662	Phase I	1	TTFields, Tadalafil, TMZ	18	AE, MDSCs change	2 years
NCT04717739	Unknown	1	TTFields	500	AE, compliance, QoL, neurocognitive function	2 years
NCT04471844	Phase IV	2	TTFields, RT, TMZ	950	OS	6 years
NCT04474353	Phase I	1	TTFields, TMZ Gadolinium, SRS	12	DLT	3 years
NCT04469075	Phase II	1	TTFields Triamcinolone Acetonide Clindamycin Phosphate	58	Grade 2 or higher skin toxicity	3 years
NCT03223103	Phase I	1	TTFields, peptides Poly‐ICLC	13	DLT	5 years
NCT05030298	Phase I, II	2	TTFields, TMZ RT, SRS	40	Toxicity	3 years
NCT05310448	Phase I	1	TTFields,	10	AE, PFS, ORR, OS	2 years
NCT05086497	Unknown	2	TTFields, MRI	155	Time to Progression	4 years
NCT04421378	Phase I, II	Multi	TTFields, RT Selinexor, TMZ lomustine, bevacizumab	474	PFS, OS, AE	3 years
NCT03642080	Unknown	1	TTFields	48	Progression of disease	5 years

Abbreviations: AE, adverse event; DLT, dose‐limiting toxicity; ICLC, lysine carboxymethylcellulose; MDSC, myeloid‐derived suppressor cell; MRI, magnetic resonance imaging; ndGBM, newly diagnosed glioblastoma; OS, overall survival; PFS, progression‐free survival; QoL, quality of life; RT, radiotherapy; SCTR, safely conducted therapy rate; SRS, stereotactic radiosurgery; TMZ, temozolomide; TTFields, tumor‐treating fields.

## 4 CONCLUSION

GBM has an extremely poor prognosis, with a recurrence rate of nearly 100% and a median survival time of 25–30 weeks after recurrence. TTField therapy has been proven to prolong PFS and OS in ndGBM patients, producing only minor local skin side effects. TTFields can interfere with the mitotic cycle of tumor cells and lead to increased apoptosis and autophagy of tumor cells. TTFields impedes DNA damage repair, which is a key mechanism in their combination with other antitumor methods. The combination of TTField‐induced BRCA1 downregulation with increased PARP cleavage produces an effect similar to that of PARP inhibitors. TTFields can induce ICD; activate T cells, DCs, and macrophages; and activate the immune microenvironment of glioma. TTFields also reduces the ability of cancer cells to metastasize and invade by downregulating multiple cytokines and destroying primary cilia. Furthermore, it interferes with TJ proteins between vascular endothelial cells, increasing BBB permeability and allowing drugs and immune cells to be distributed more evenly throughout the tumor. In clinical applications, the new Stupp protocol has demonstrated excellent survival benefits. Whether it is safe and feasible to advance the time of application of TTFields to a time concurrent with chemoradiotherapy has been preliminarily explored. After proper adjustment of the PTV and scalp protection, wearing electrode patches during radiotherapy is safe and feasible. In addition, TTField therapy shows potential in combination with immune checkpoint inhibitors, tumor vaccines, antimitotic drugs, and PARP inhibitors as well as the potential for application in patients with subtentorial glioma. With further reduction of treatment cost in the future, more patients will benefit from TTField treatment. Thus, research is still needed to explore new ways to combine TTFields with other therapies and optimize the use of TTFields to realize its full potential in ndGBM patients.

## FUNDING INFORMATION

This work was supported by the National Natural Science Foundation of China (No. 82102844) and the Natural Science Foundation of Liaoning Province (2022‐MS‐193).

## CONFLICT OF INTEREST STATEMENT

The authors declare that there is no conflict of interest.

## Data Availability

Data sharing not applicable to this article as no datasets were generated or analysed during the current study.
